# Supplementary material for: Local rabies transmission and regional spatial coupling in European foxes
Source: PLoS One. 2020 May 29;15(5):e0220592. doi: 10.1371/journal.pone.0220592 (PMC7259497; doi:10.1371/journal.pone.0220592)
Supplement: S1 Fig — Posterior and prior distributions and traceplots for all parameters in the model. (PDF) [file pone.0220592.s001.pdf]

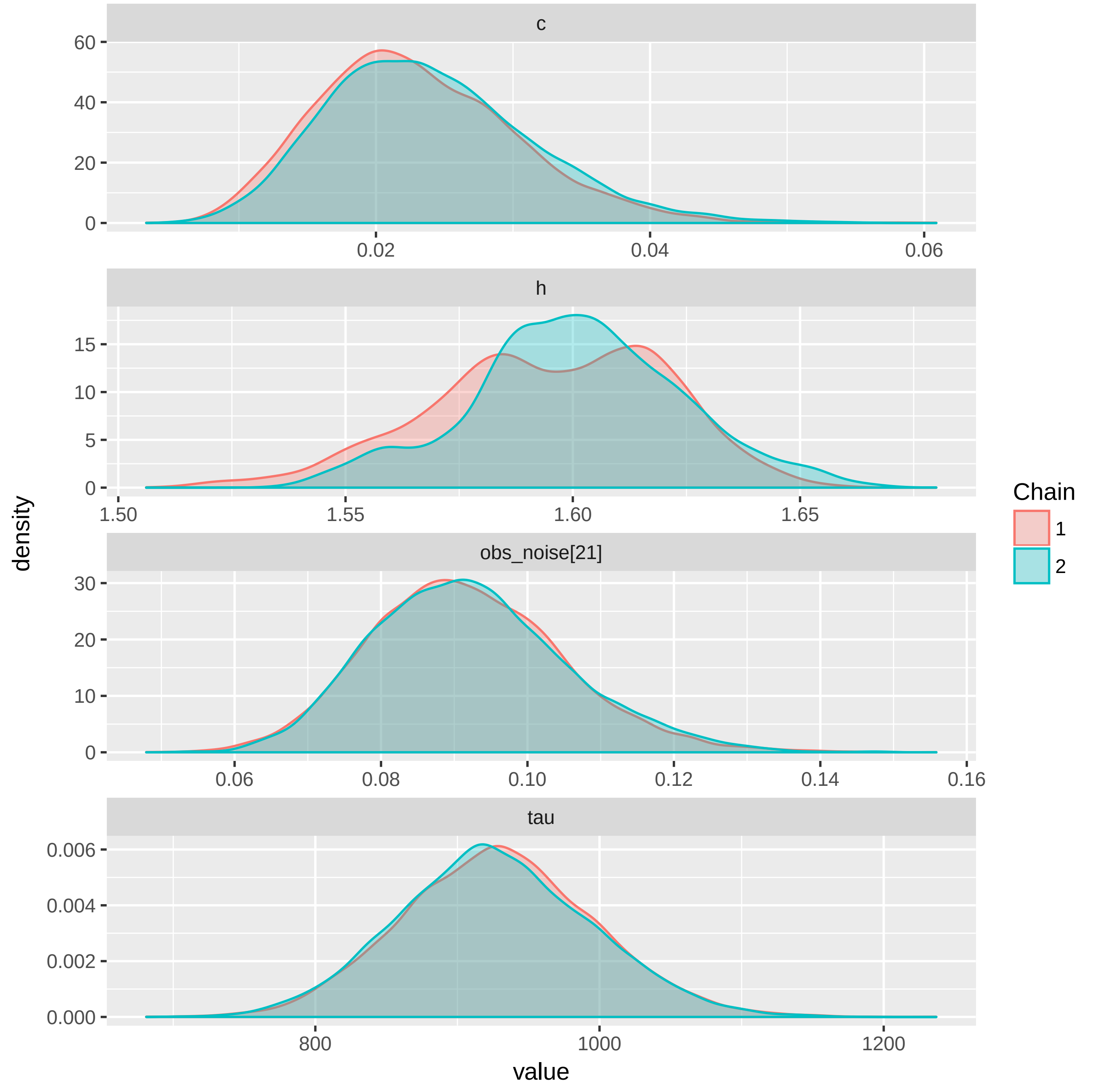
c
60
40
20
0
0.02
0.04
0.06
h
15
10
5
0
1.50
1.55
1.60
1.65
obs_noise[21]
30
20
10
0
0.06
0.08
0.10
0.12
0.14
0.16
tau
0.006
0.004
0.002
0.000
800
1000
1200
density
value
Chain
1
2

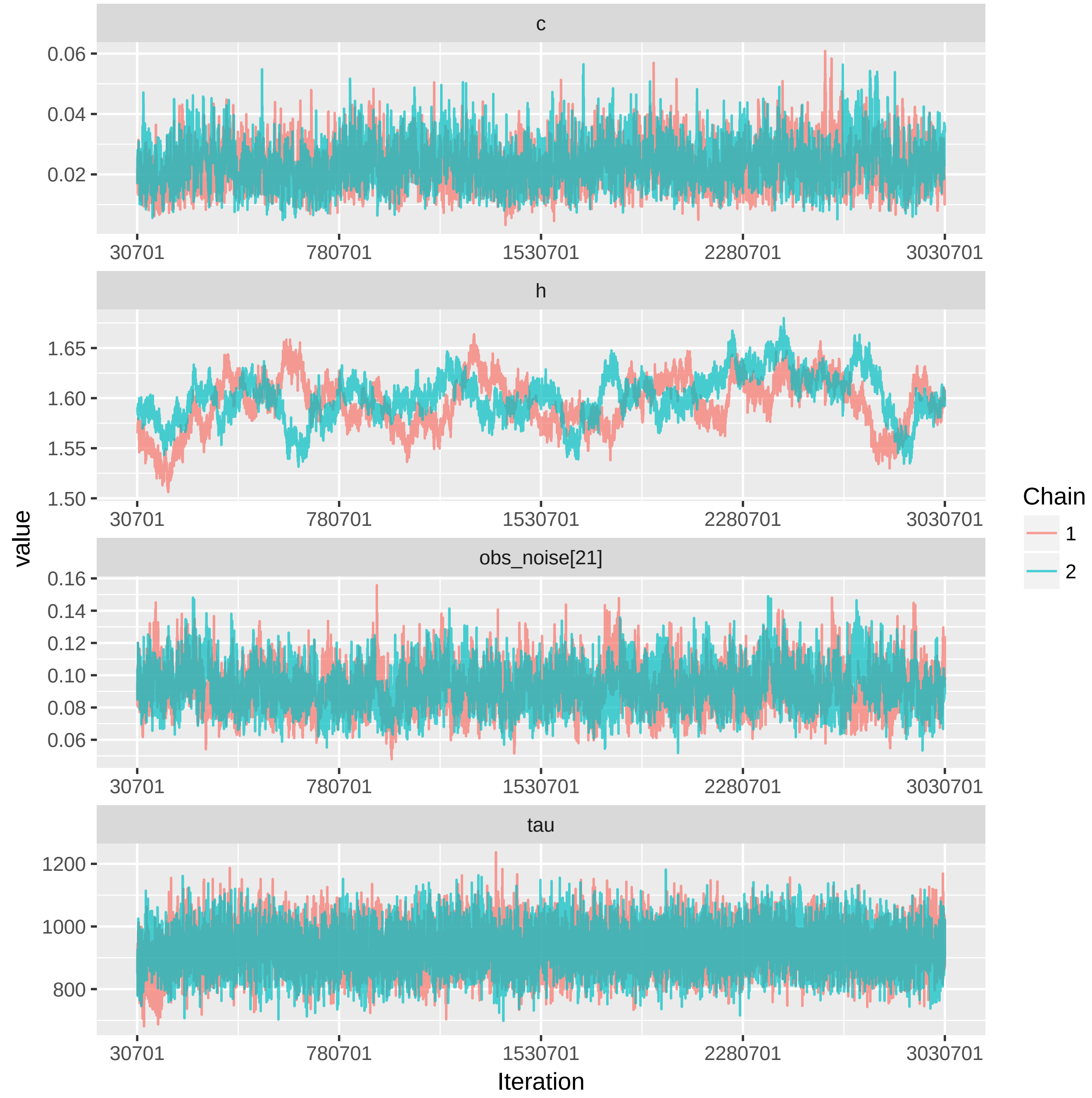
c
h
obs_noise[21]
tau
value
Iteration
Chain
1
2
0.06
0.04
0.02
1.65
1.60
1.55
1.50
0.16
0.14
0.12
0.10
0.08
0.06
1200
1000
800
30701
780701
1530701
2280701
3030701

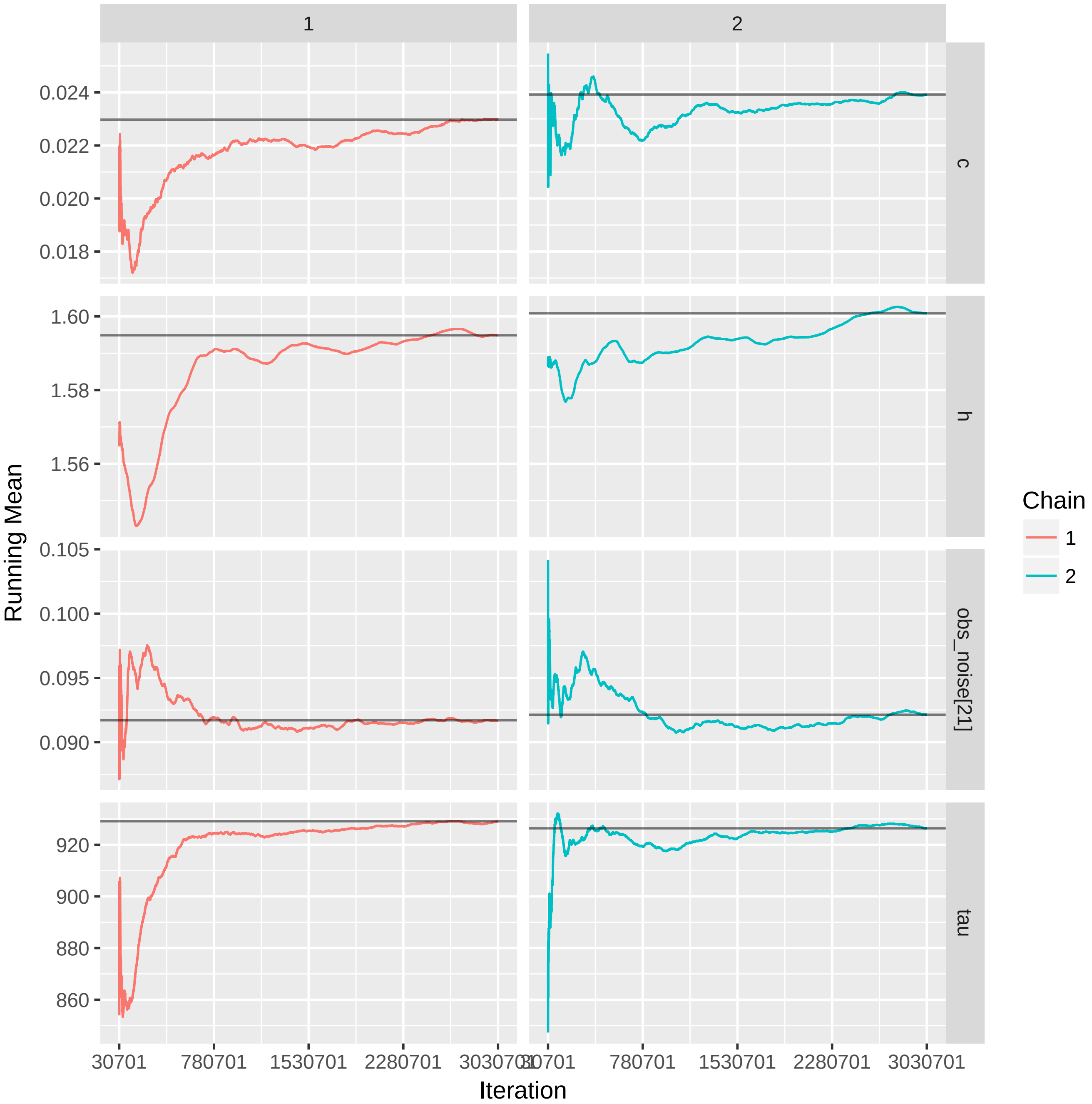
1
2
c
h
obs_noise[21]
tau
0.024
0.022
0.020
0.018
1.60
1.58
1.56
0.105
0.100
0.095
0.090
920
900
880
860
30701
780701
1530701
2280701
3030701
Running Mean
Iteration
Chain
1
2

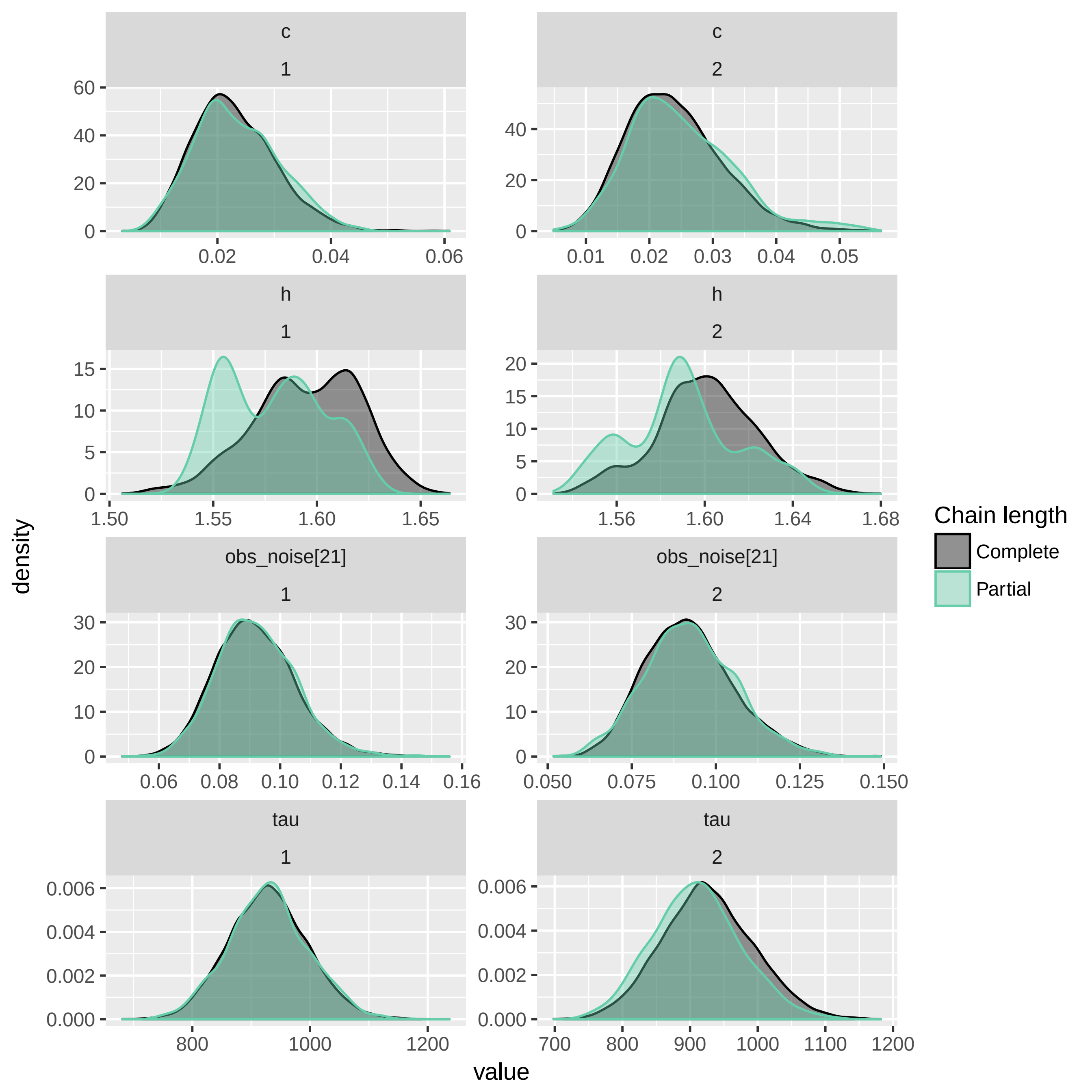

c
1
c
2
h
1
h
2
obs_noise[21]
1
obs_noise[21]
2
tau
1
tau
2
density
value
Chain length
Complete
Partial

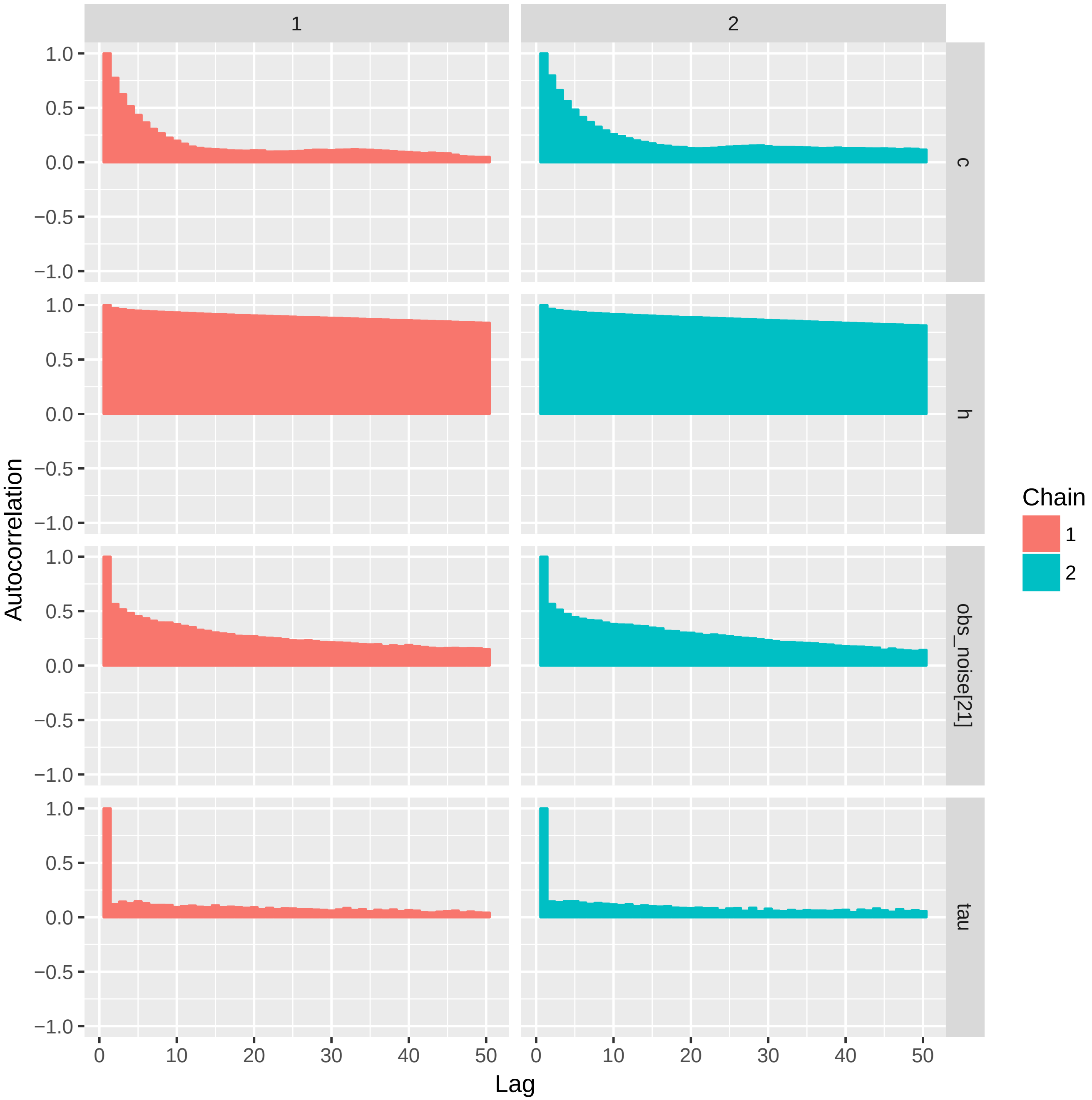

1
2
c
h
obs_noise[21]
tau
Autocorrelation
1.0
0.5
0.0
−0.5
−1.0
0
10
20
30
40
50
Lag
Chain
1
2

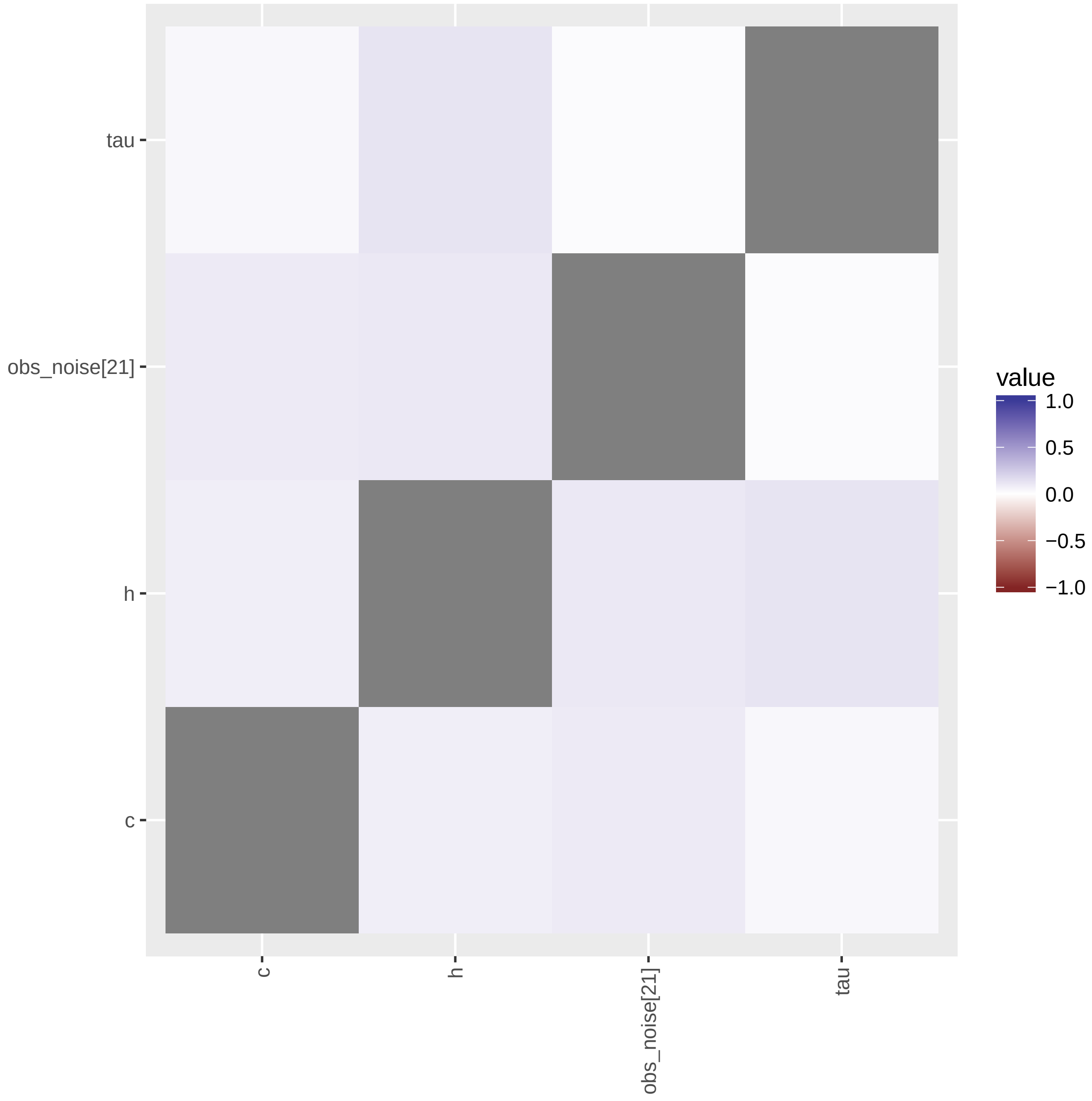
tau
obs_noise[21]
h
c
c
h
obs_noise[21]
tau
value
1.0
0.5
0.0
−0.5
−1.0

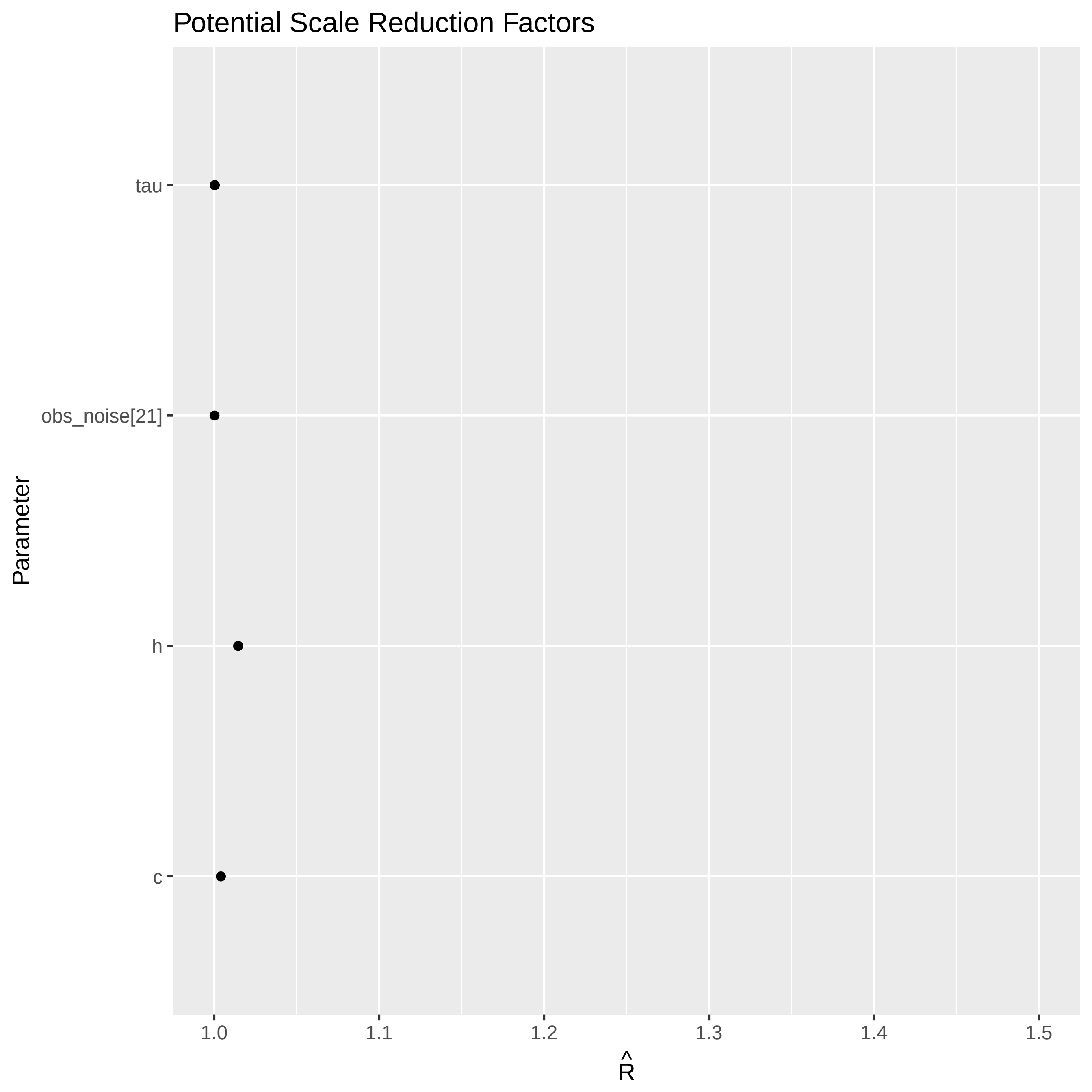

Potential Scale Reduction Factors
tau
obs_noise[21]
h
c
Parameter
1.0
1.1
1.2
1.3
1.4
1.5
R̂

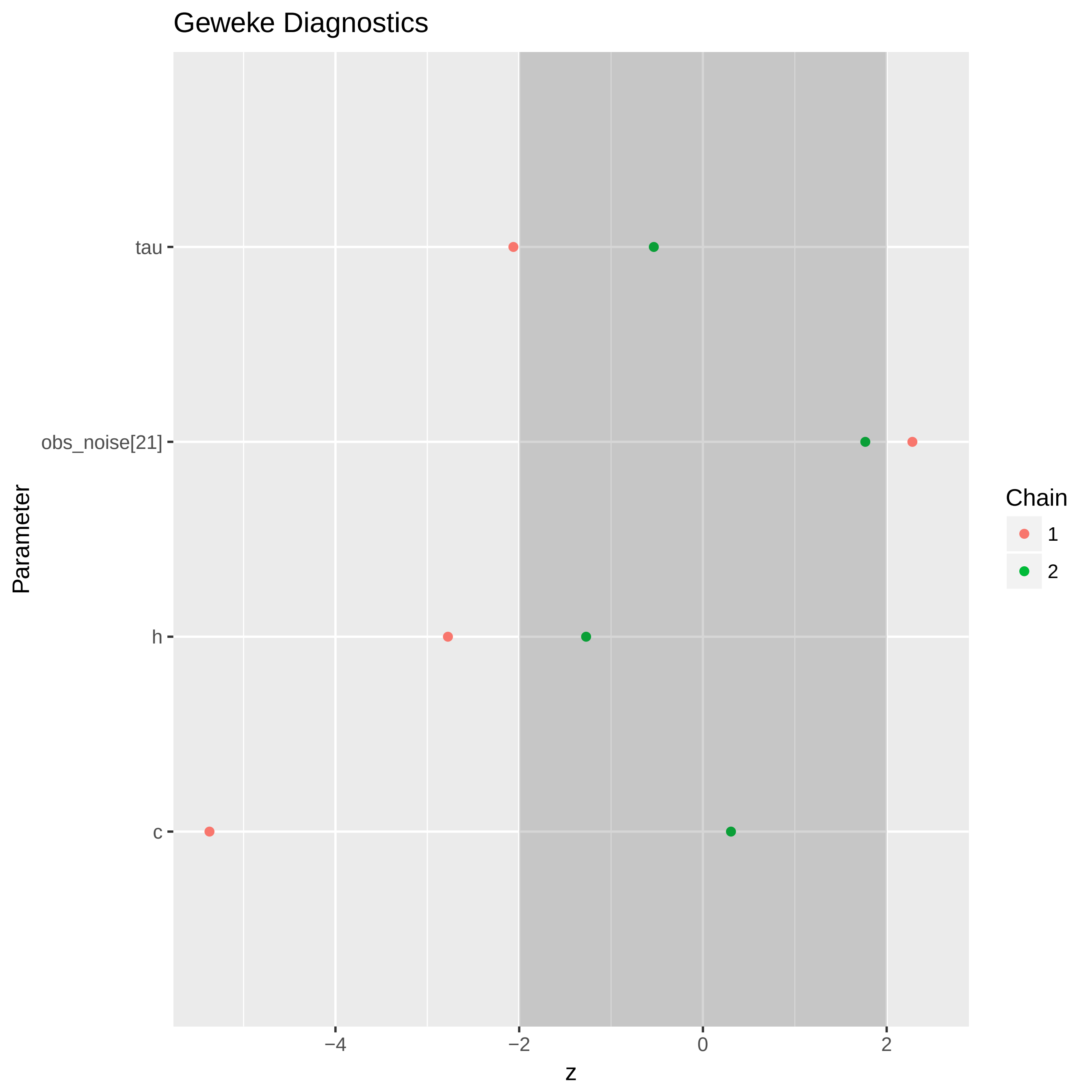

Geweke Diagnostics
tau
obs_noise[21]
h
c
Parameter
−4
−2
0
2
z
Chain
1
2
