# Supplementary material for: Local rabies transmission and regional spatial coupling in European foxes
Source: PLoS One. 2020 May 29;15(5):e0220592. doi: 10.1371/journal.pone.0220592 (PMC7259497; doi:10.1371/journal.pone.0220592)
Supplement: S2 Fig — Posterior and prior distributions and traceplots for all parameters in the model when the observation rate θ was given a wide prior. (PDF) [file pone.0220592.s002.pdf]

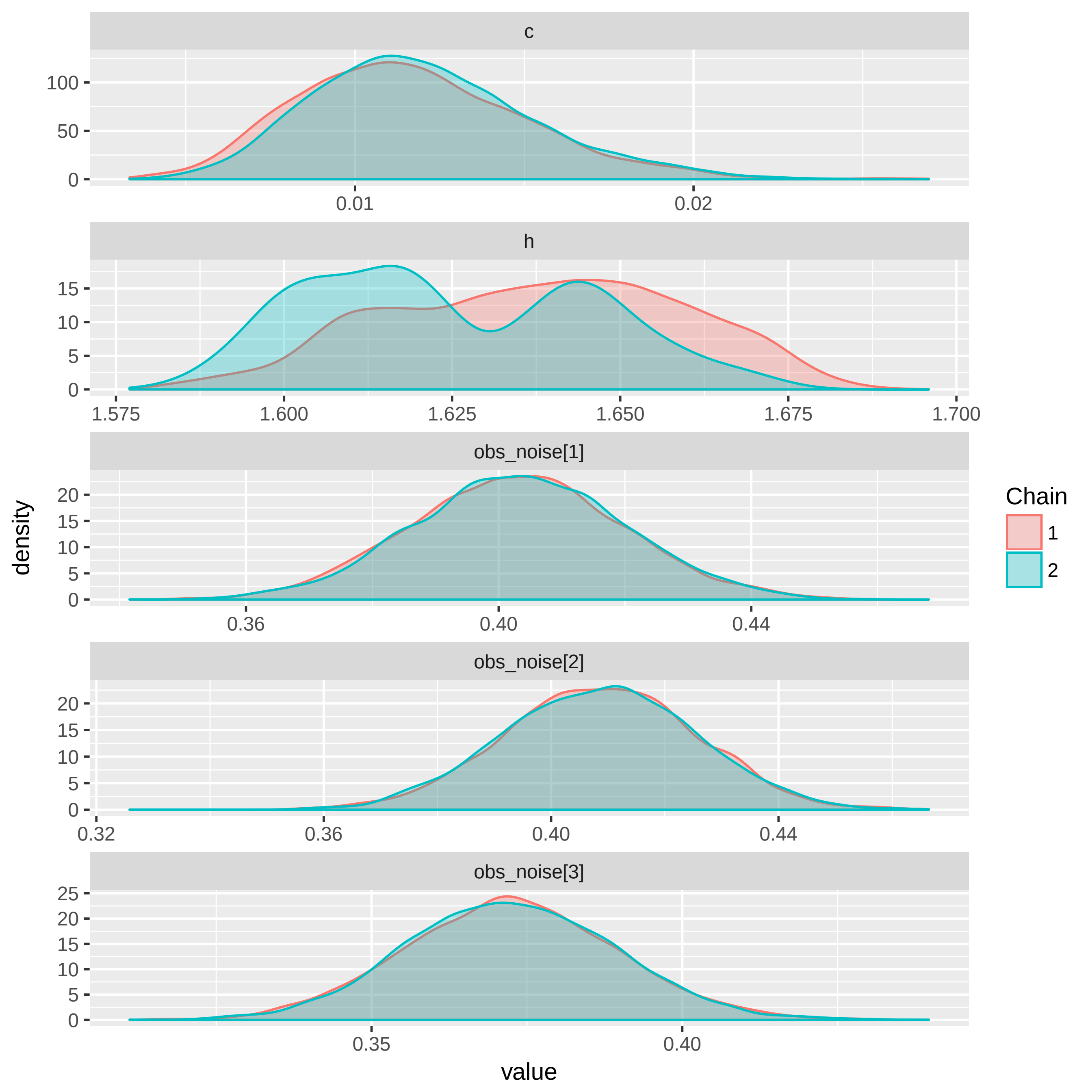

c
100
50
0
0.01
0.02
h
15
10
5
0
1.575
1.600
1.625
1.650
1.675
1.700
obs_noise[1]
20
15
10
5
0
0.36
0.40
0.44
obs_noise[2]
20
15
10
5
0
0.32
0.36
0.40
0.44
obs_noise[3]
25
20
15
10
5
0
0.35
0.40
density
value
Chain
1
2

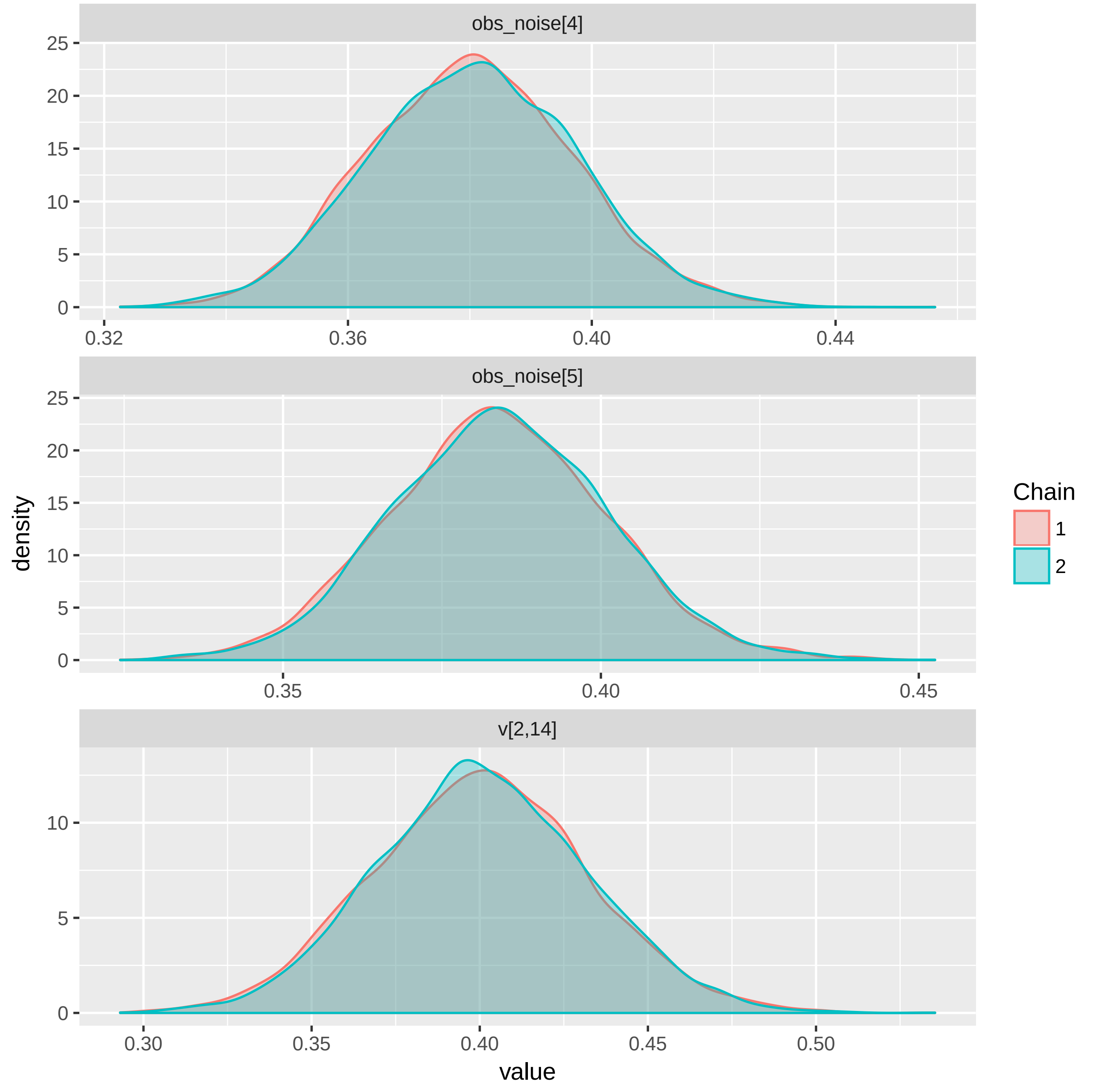
obs_noise[4]
obs_noise[5]
v[2,14]
density
value
Chain
1
2
0.32
0.36
0.40
0.44
0.35
0.40
0.45
0.30
0.35
0.40
0.45
0.50
0
5
10
15
20
25

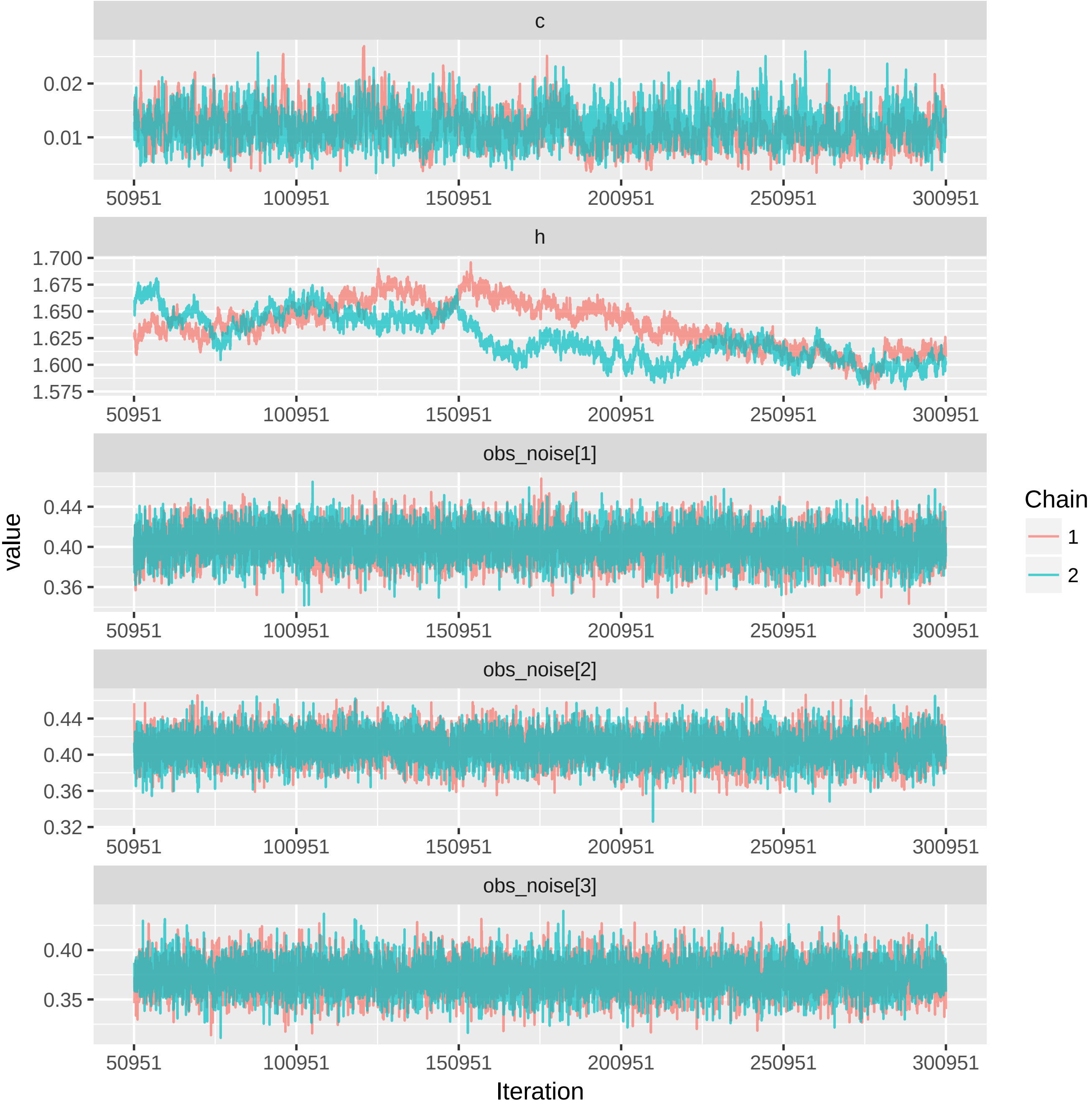
c
0.02
0.01
h
1.700
1.675
1.650
1.625
1.600
1.575
obs_noise[1]
0.44
0.40
0.36
obs_noise[2]
0.44
0.40
0.36
0.32
obs_noise[3]
0.40
0.35
50951
100951
150951
200951
250951
300951
Iteration
value
Chain
1
2

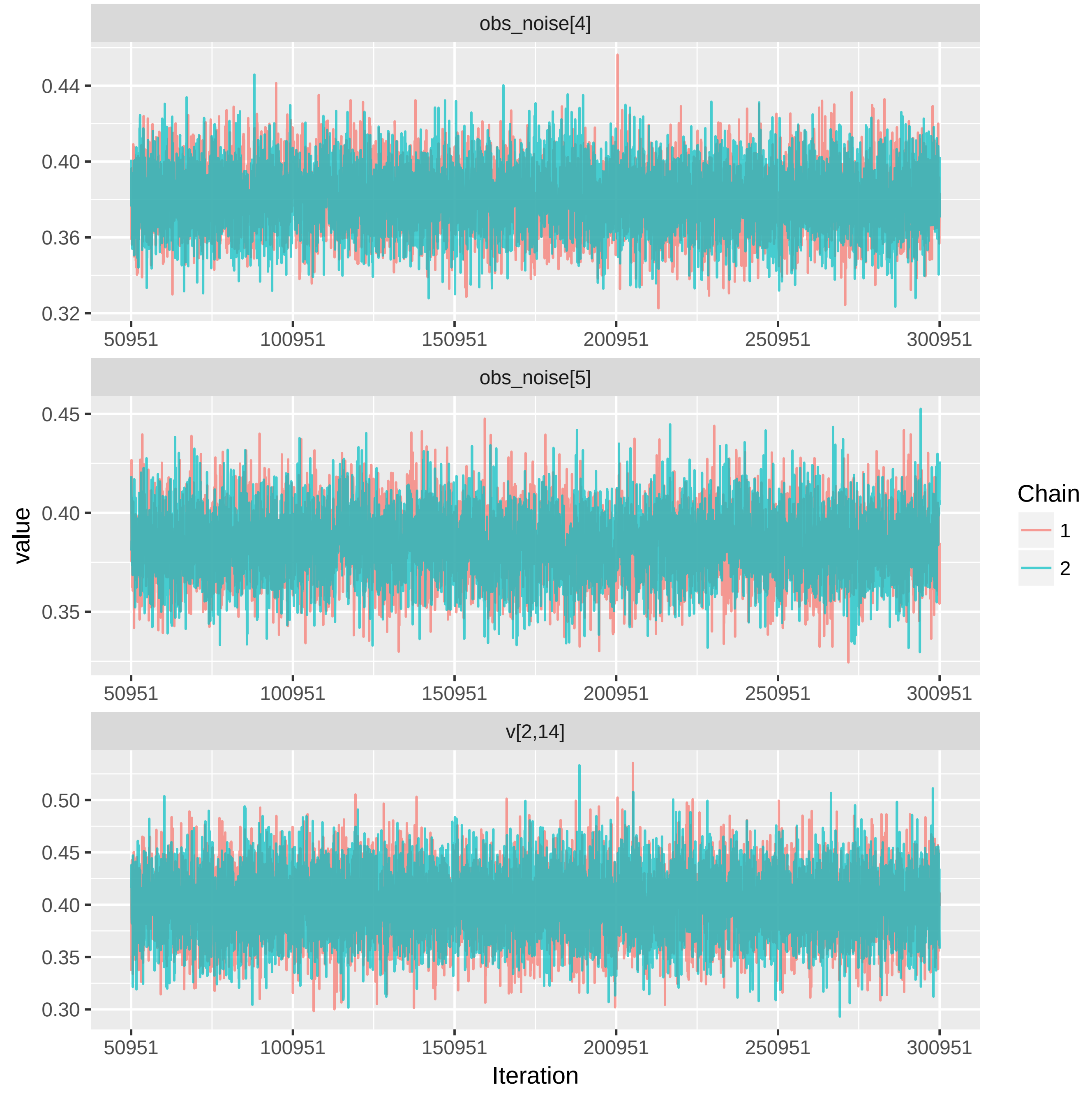
obs_noise[4]
obs_noise[5]
v[2,14]
value
Iteration
Chain
1
2
50951
100951
150951
200951
250951
300951
0.44
0.40
0.36
0.32
0.45
0.35
0.50
0.30

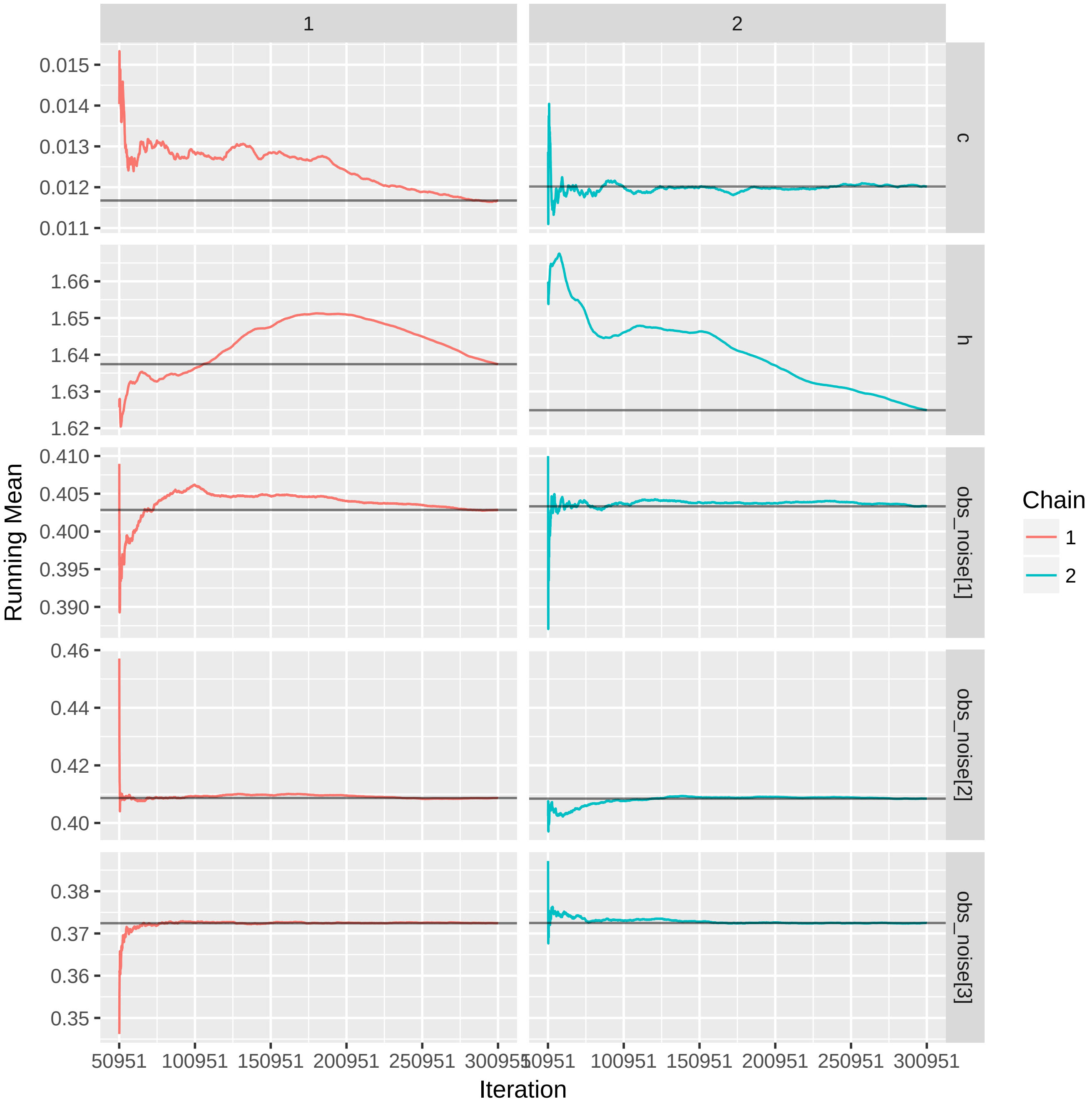

1
2
c
h
obs_noise[1]
obs_noise[2]
obs_noise[3]
0.015
0.014
0.013
0.012
0.011
1.66
1.65
1.64
1.63
1.62
0.410
0.405
0.400
0.395
0.390
0.46
0.44
0.42
0.40
0.38
0.37
0.36
0.35
Running Mean
50951
100951
150951
200951
250951
300951
Iteration
Chain
1
2

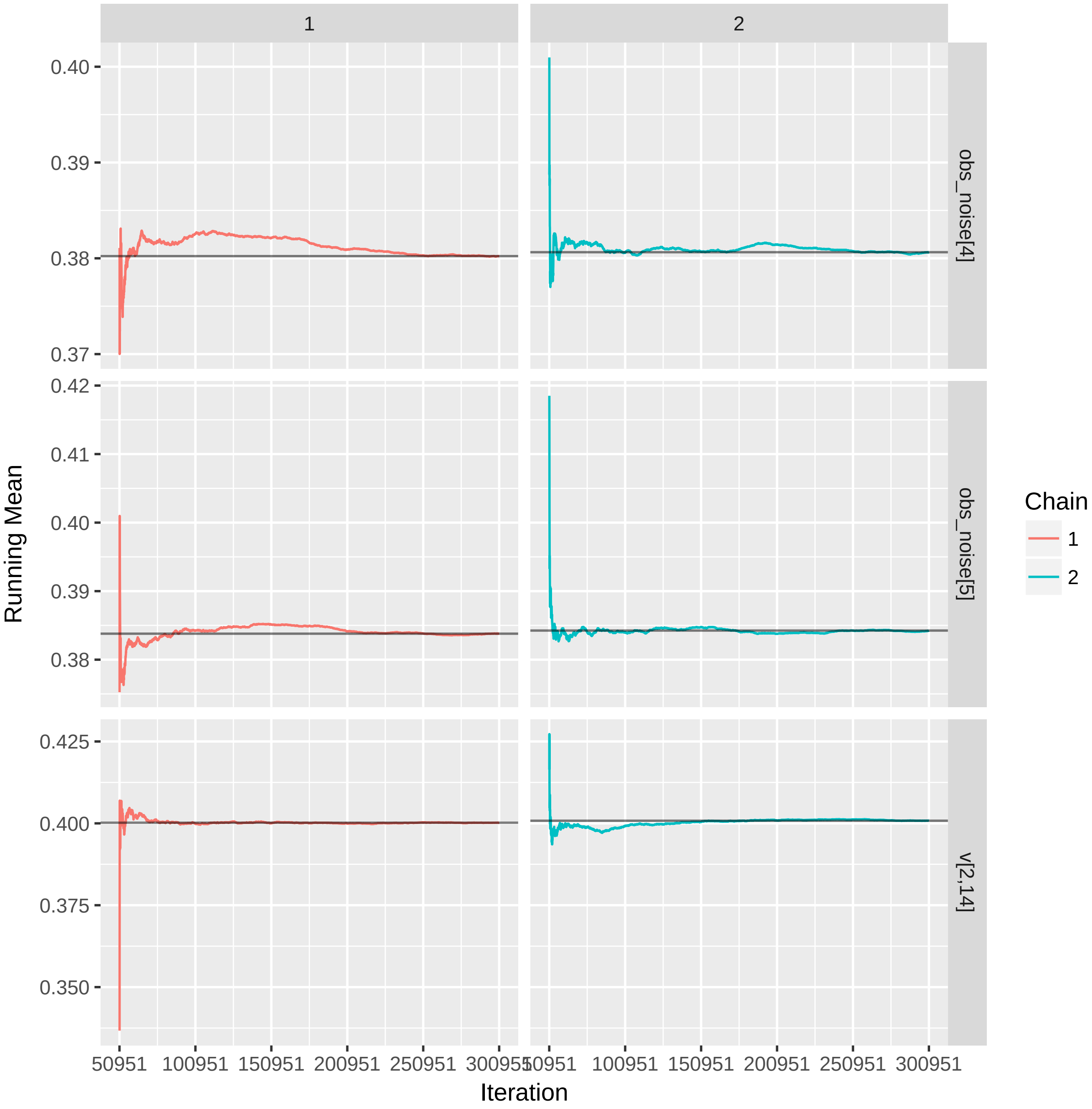

1
2
0.40
0.39
0.38
0.37
0.42
0.41
0.40
0.39
0.38
0.425
0.400
0.375
0.350
obs_noise[4]
obs_noise[5]
v[2,14]
50951
100951
150951
200951
250951
300951
Iteration
Running Mean
Chain
1
2

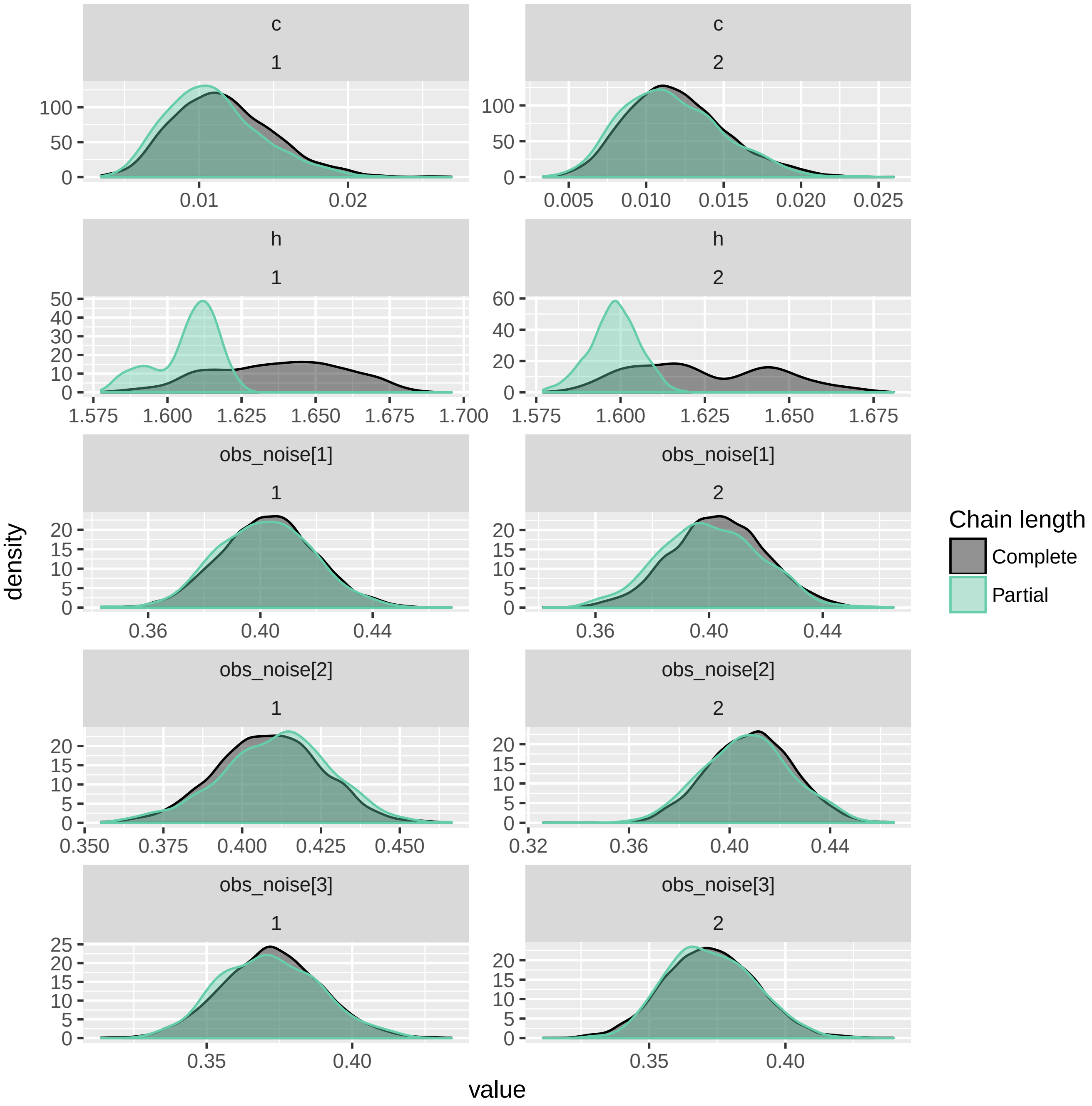

c
1
c
2
h
1
h
2
obs_noise[1]
1
obs_noise[1]
2
obs_noise[2]
1
obs_noise[2]
2
obs_noise[3]
1
obs_noise[3]
2
density
value
Chain length
Complete
Partial

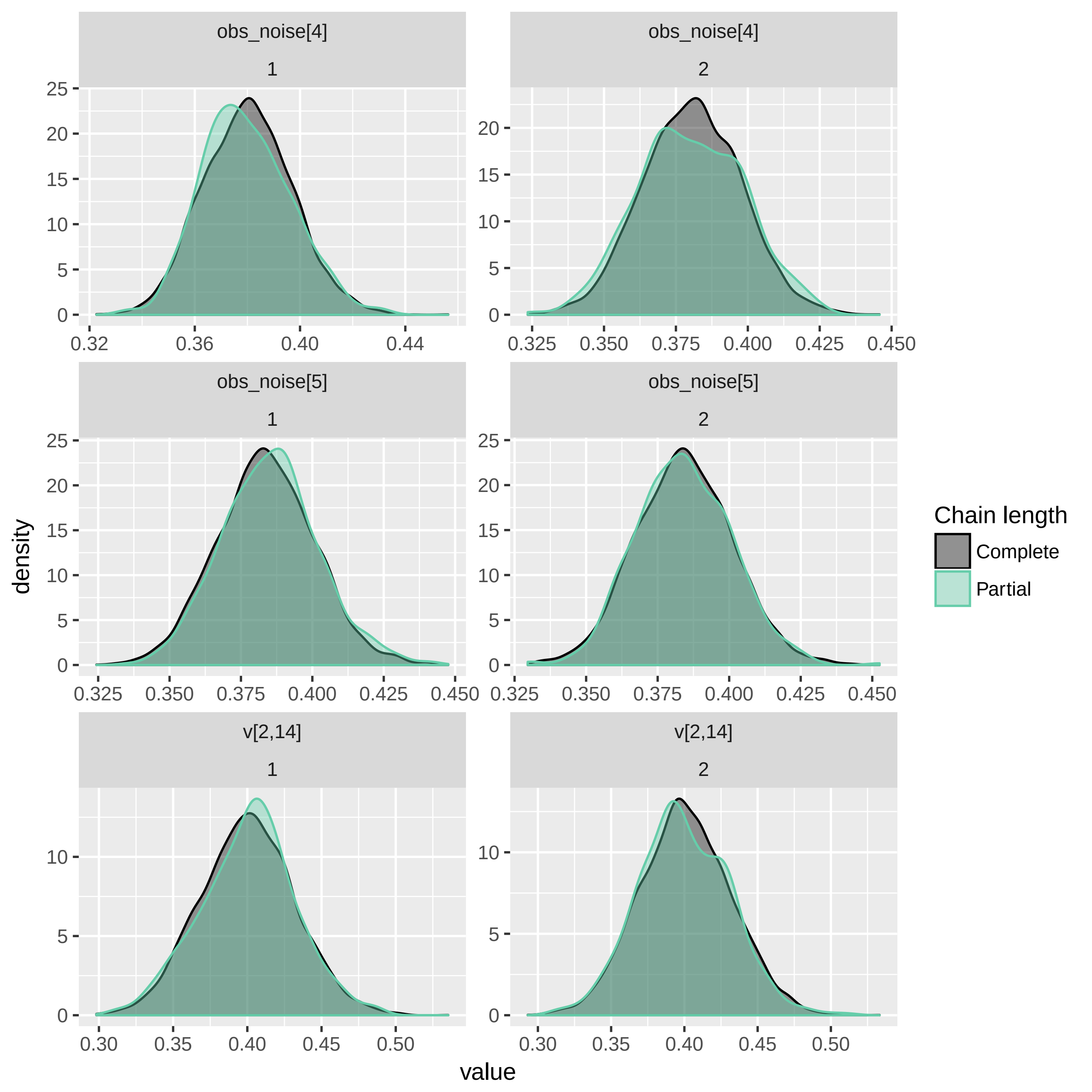
obs_noise[4]
1
obs_noise[4]
2
obs_noise[5]
1
obs_noise[5]
2
v[2,14]
1
v[2,14]
2
density
value
Chain length
Complete
Partial

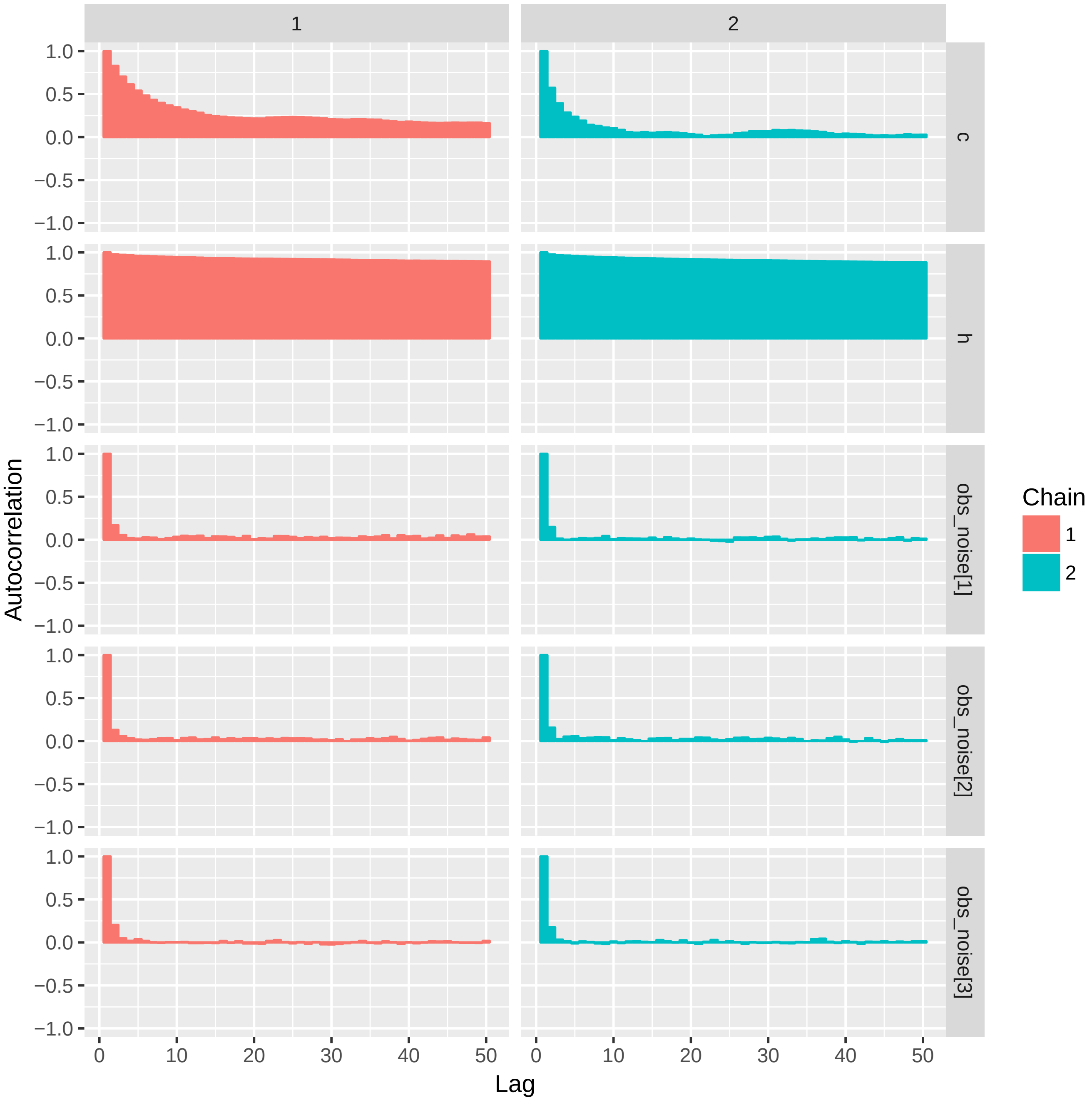
1
2
c
h
obs_noise[1]
obs_noise[2]
obs_noise[3]
1.0
0.5
0.0
−0.5
−1.0
0
10
20
30
40
50
Autocorrelation
Lag
Chain
1
2

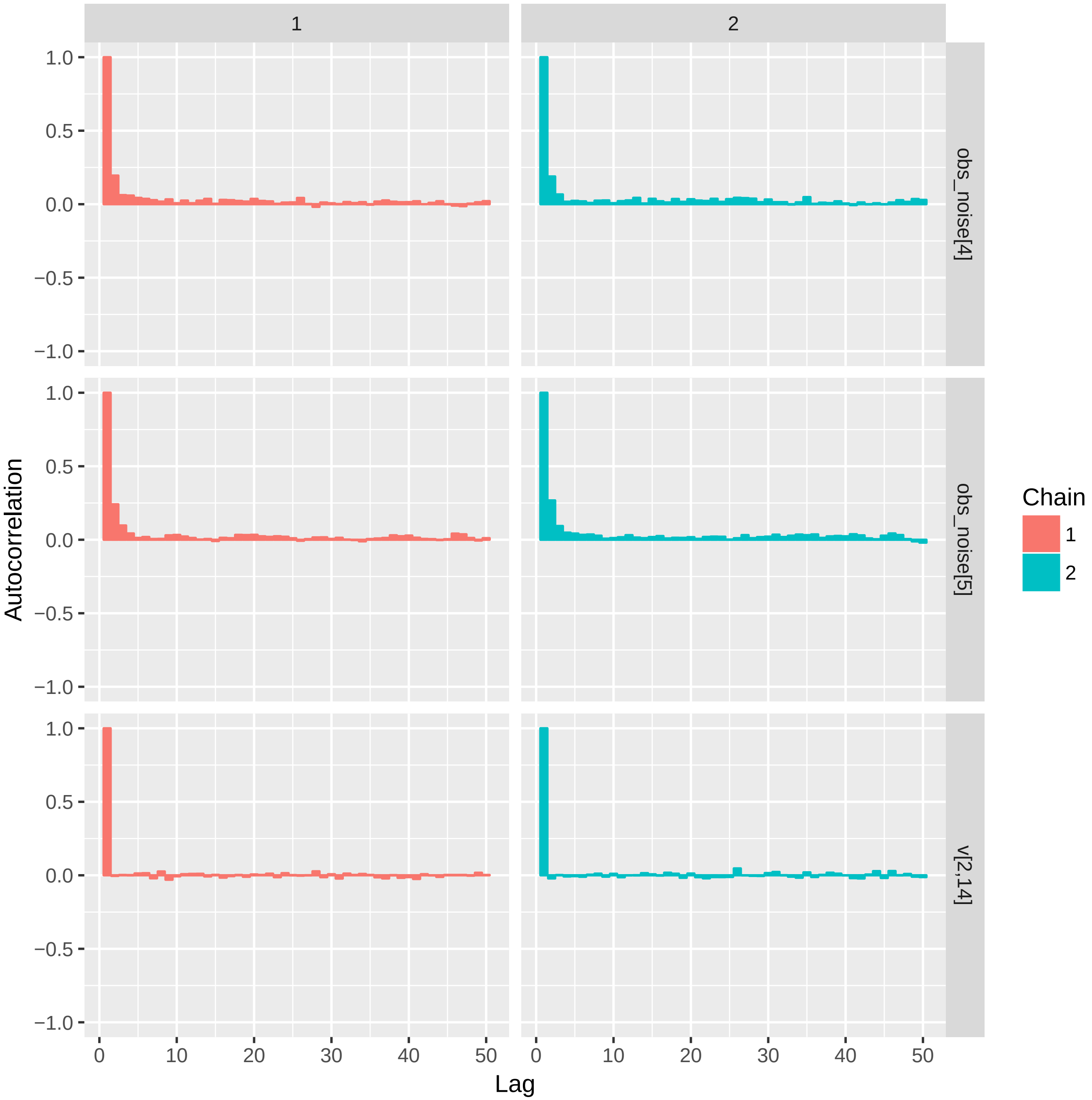

1
2
obs_noise[4]
obs_noise[5]
v[2,14]
Autocorrelation
Lag
Chain
1
2

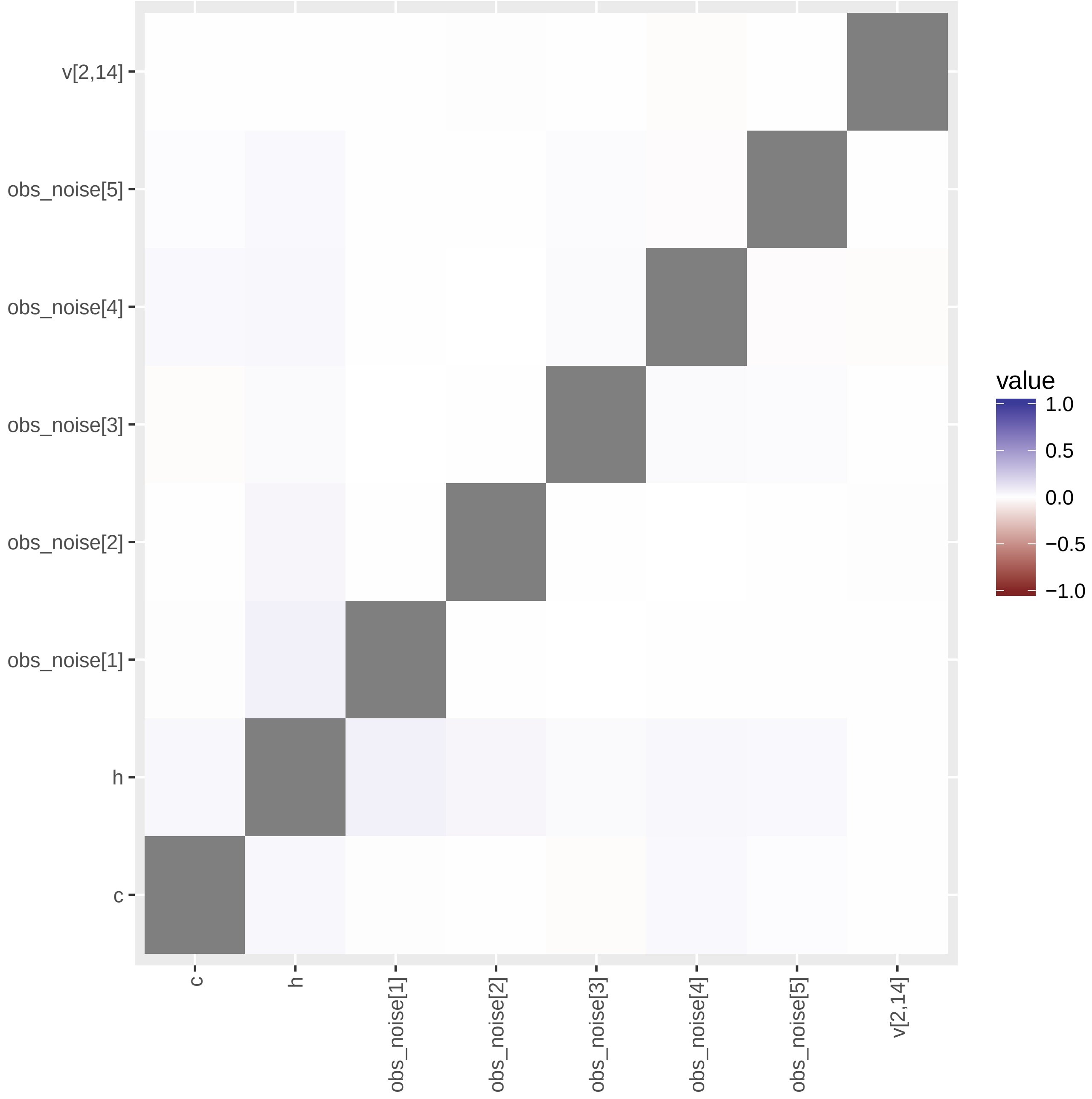
v[2,14]
obs_noise[5]
obs_noise[4]
obs_noise[3]
obs_noise[2]
obs_noise[1]
h
c
value
1.0
0.5
0.0
−0.5
−1.0

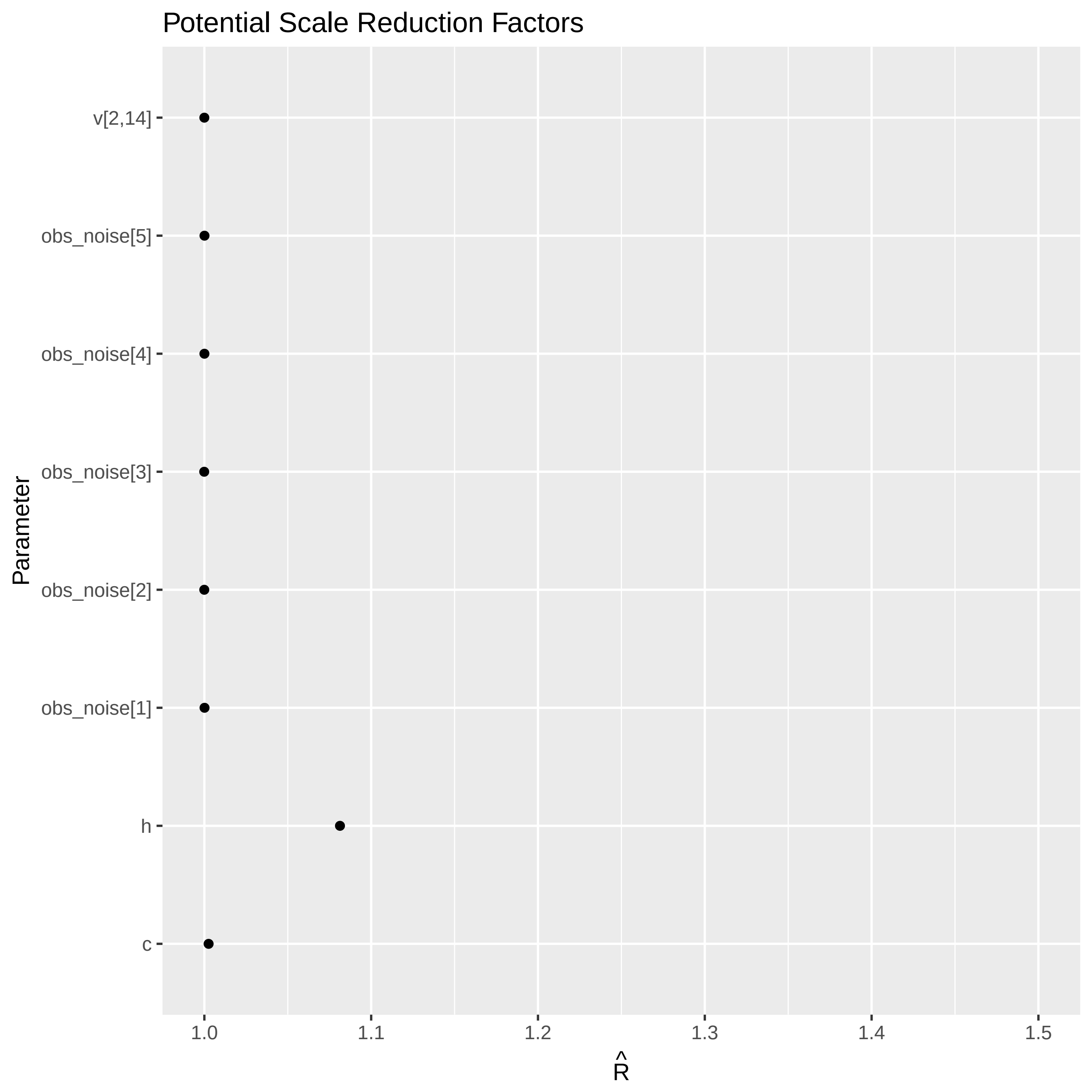

Potential Scale Reduction Factors
v[2,14]
obs_noise[5]
obs_noise[4]
obs_noise[3]
obs_noise[2]
obs_noise[1]
h
c
Parameter
1.0
1.1
1.2
1.3
1.4
1.5
$\hat{R}$

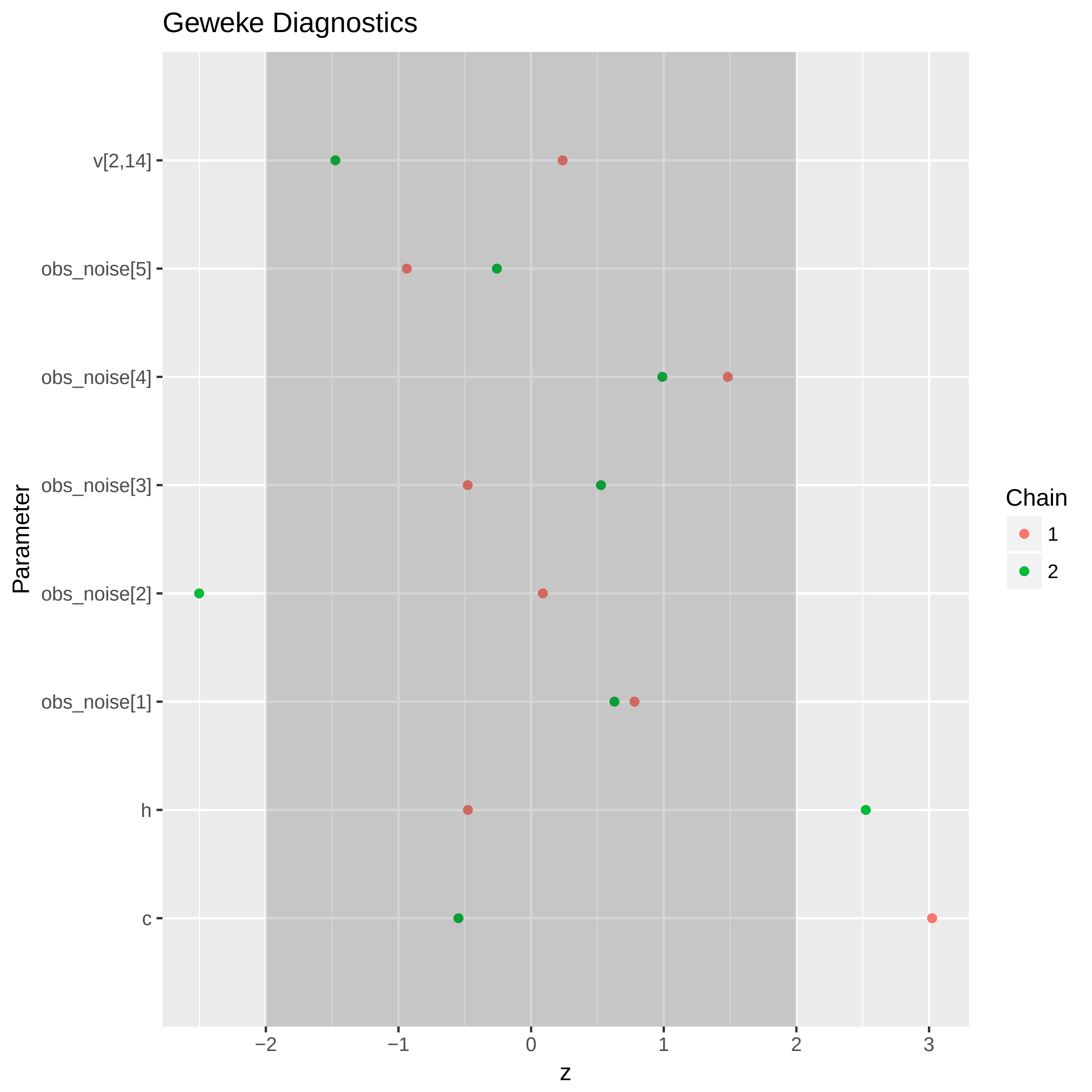
Geweke Diagnostics
v[2,14]
obs_noise[5]
obs_noise[4]
obs_noise[3]
obs_noise[2]
obs_noise[1]
h
c
Parameter
−2
−1
0
1
2
3
z
Chain
1
2

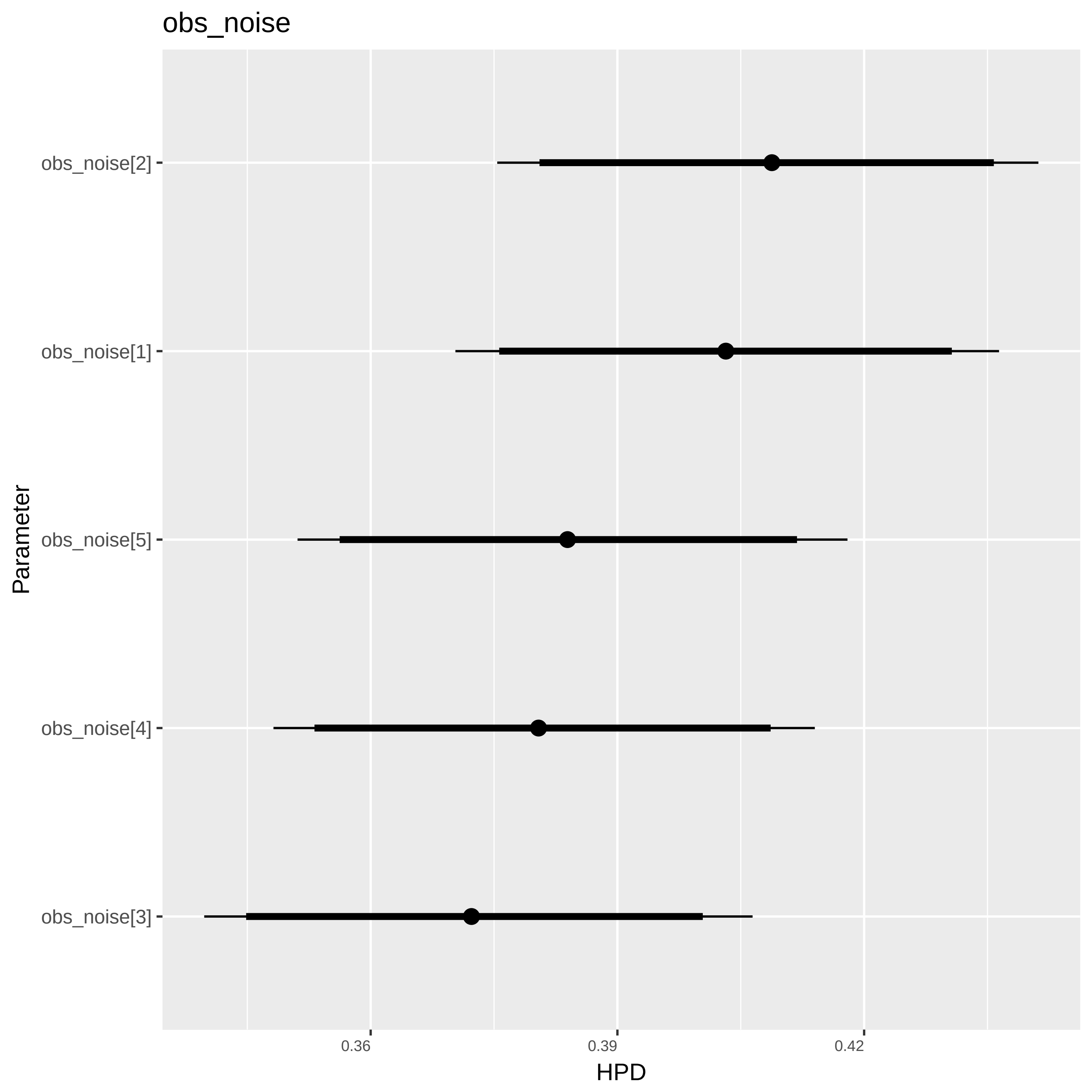

obs_noise
obs_noise[2]
obs_noise[1]
obs_noise[5]
obs_noise[4]
obs_noise[3]
Parameter
0.36
0.39
0.42
HPD
